# Lingual Dystonia Following Thalamic Infarction in a Patient on Methotrexate Therapy for Hidradenitis Suppurativa

**DOI:** 10.7759/cureus.82974

**Published:** 2025-04-25

**Authors:** Iva Sarac, Fran Borovecki, Liborija Lugovic Mihic, Hanna Pasic, Helena Sarac

**Affiliations:** 1 Department of Neurology, University of Zagreb, Faculty of Medicine, Zagreb, HRV; 2 Department of Neurology, University Hospital Centre Zagreb, Zagreb, HRV; 3 Department of Dermatovenereology, University of Zagreb, Faculty of Dental Medicine, Zagreb, HRV; 4 Department of Psychiatry, University Hospital Centre "Sestre milosrdnice", Zagreb, HRV

**Keywords:** adalimumab (humira), hidradenitis suppurativa (hs), lingual dystonia, oral methotrexate, thalamic infarction

## Abstract

Lingual dystonia is a rare form of focal dystonia involving involuntary, repetitive, and often painful muscle contractions of the tongue, which can lead to abnormal tongue posturing or protrusion, dysarthria, and/or dysphagia. Lingual dysotnia can be primary or secondary to various conditions. The thalamus is a key relay center between the motor cortex and basal ganglia, and damage here can disrupt motor signaling pathways, possibly leading to focal dystonia, like lingual dystonia. Methotrexate (MTX) can cause acute to subacute neurological complications, which are linked with high doses of MTX, while chronic MTX neurotoxicity develops more slowly, resulting in persistent focal neurological deficits. We present a 56-year-old male patient on MTX therapy who developed an isolated lingual dystonia as a chronic presentation of a small infarction in the posterolateral thalamus. The finding of thalamic infarction on brain CT and the lack of clinical improvement in dystonia symptoms after prolonged MTX withdrawal from therapy indicate that lingual dystonia could be directly causally related to thalamic insult, while it is less likely that lingual dystonia is a side effect of MTX therapy. This case provides us with new knowledge in determining the etiology of lingual dystonia in the light of thalamic infarction and MTX neurotoxicity.

## Introduction

Lingual dystonia is a rare focal dystonia, with a prevalence of 4% [1]. Lingual dystonia is a variant of oromandibular dystonia, characterized by rhythmic or tremor-like involuntary movements of the tongue, often in the form of task-specific contractions of the tongue muscle triggered by speech or eating. Lingual dystonia may be primary or secondary to various conditions, including vascular, autoimmune, infectious, and drug-related [1-3]. The thalamus is a key relay center for neural pathways, including cortico-striato-thalamo-cortical and cortico-ponto-cerebello-thalamo-cortical loops involved in sensory, motor, and cognitive functions. Lesions in this region may cause focal abnormal involuntary movements, including delayed-onset dystonia [3]. The basal ganglia interfere with movements by balancing excitation and inhibition within the thalamo-cortical circuits. Isolated lingual dystonia is extremely rare after acute and/or chronic stroke, and infarctions are usually seen in the basal ganglia, subcortical white matter, thalamus, and cerebellum, although most patients almost always have additional ischemic lesions bilaterally [4-6]. Methotrexate (MTX) can cause acute (seizures, headaches, stroke-like episodes), subacute, and long-term neurotoxicity (cognitive impairments and gradual onset of focal weakness). In adults, the neurotoxicity rate is 20% for intrathecal administration, 1.13% for intravenous administration, and 3.39% for combined intrathecal and intravenous administration of MTX [7]. Acute to subacute neurological complications have been linked with high doses and multiple treatments of MTX. Chronic MTX-induced leukoencephalopathy develops more slowly and ultimately results in persistent focal neurological deficits [8]. Lingual dystonia should be considered as a potential side effect of MTX therapy if there is no other apparent cause of lingual dystonia. Except for one-single-center cohort study [7], one case study [8], and one prospective cohort study which provided data on neurotoxicity and safety of intrathecal and high-dose MTX in childhood acute lymphoblastic leukemia [9], detailed data on the prevalence, clinical manifestations, and risk factors for MTX-induced neurological complications are limited, especially those resulting from oral administration of MTX.

This study aimed to detail lingual dystonia following chronic infarction in the right posterolateral thalamus in one patient on oral MTX. Although detection of thalamic infarction should rule out MTX neurotoxicity as a cause of lingual dystonia, MTX therapy still complicates the final diagnosis in this patient, making it challenging to determine the primary cause of lingual dystonia.

## Case presentation

**Figure 1:**
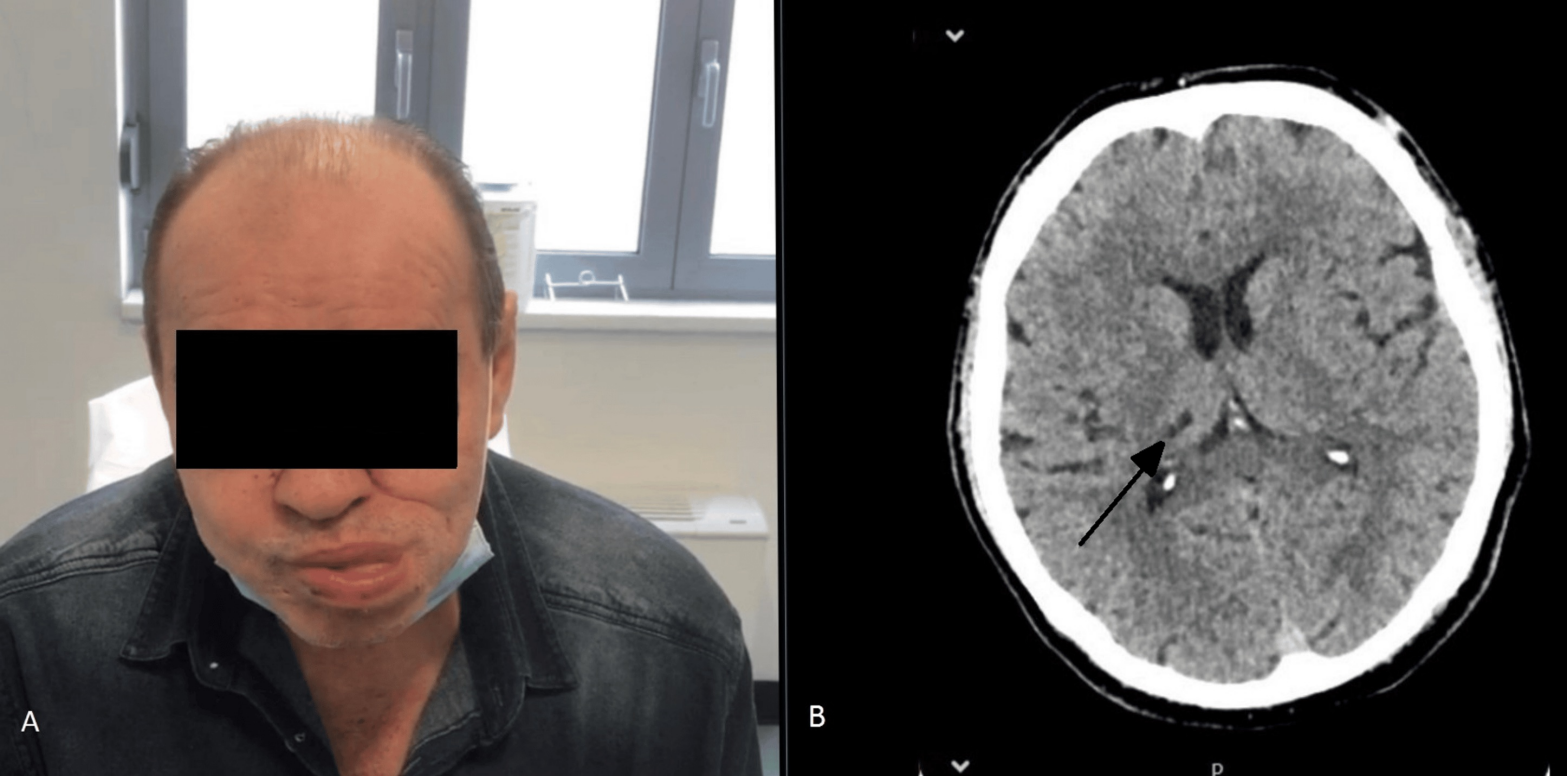
(A) Clinical image showing lingual dystonia. (B) Axial image created from MSCT scan showing chronic infarction in the right posterolateral thalamus.

A 56-year-old man was referred to the Neurology Department for evaluation and management of lingual dystonia, which had been evolving for two years. He has had a long history of diabetes mellitus (DM) type II and hypertension. He smoked 20 cigarettes per day. As part of his relevant medical history, he was diagnosed with hidradenitis suppurativa 10 years ago. In the patient's medical history, he reported involuntary tongue movements at rest. During the physical examination, the patient manifested intermittent abnormal involuntary movements of the tongue to the left side at rest, which were abolished by tongue protrusion or during speech (Figure 1A). He had no problems with speech or swallowing. There was no other focal neurological deficit. Brain magnetic source CT (MSCT) detected a small chronic infarction in the right posterolateral thalamus, and no other ischemic lesions were found (Figure 1B).

Cerebral CT angiogram showed calcified atherosclerotic plaques of the aortic arch and at the origins of the supra-aortic branches and both vertebral arteries without significant stenoses. Both vertebral arteries are monitored continuously in the cervical and intracranial segments. Marginal calcified plaques of the origin of both anterior coronary interarterial vessels (ACIs) were visible, causing stenosis of up to 30%. No signs of vasculitis were described. Brain MRI was not performed due to the presence of shrapnel in his body.

Physical examination did not detect a skin abscess. Examination of the oral cavity revealed oral dryness. He was treated several times with peroral antibiotics and topical resorcinol until September 2018, when at the Department of Dermatology and Venereology adalimumab was introduced in doses of doses of 160 mg at week 0.80 mg at week 2, and 40 mg each week thereafter, in addition to local therapy with 10% resorcinol solution at the location of the skin changes. After 10 months, despite the improvement of dermatoses and Physician’s Global Assessment of Clinical Condition (PGA) staging, biological therapy was discontinued due to recurrent septic conditions. Since March 2020, he has been on oral MTX 25 mg sc once a week and oral prednisone 10 mg daily. Complete blood count revealed elevated erythrocyte sedimentation rate (ESR) of 79 mm/h, hemoglobin (Hb) 111 g/l, leukocytes 15.4×10^9^/l, with neutrophils 81.7%. Standard biochemistry was within normal limits. Immunological tests showed the presence of antinuclear antibodies (ANAs), Golgi-type (AC-22* +), positive anti-U1-RNP antibodies (154 AU/ml), while rheumatoid factor, antineutrophil cytoplasmic antibodies (ANCA), and anticardiolipin antibodies (ACA) were negative. A comprehensive metabolic panel that measures glucose level, electrolyte and fluid balance, kidney function, liver function, and thyroid function was within normal ranges Table 1.

**Table 1:** Soluble markers of inflammation and autoantibodies in patients with lingual dystonia and hidradenitis suppurativa. PV: photovoltaic; APTV: arc thermal performance value; TSH: thyroid-stimulating hormone; TPO: thyroid peroxidase; ANA: antinuclear antibodies; U1RNP: U1 ribonucleoprotein; ANCA: antineutrophil cytoplasmic antibodies The presence of inflammatory markers (raised ERS, CRP, leukocytosis) and autoimmune markers (ANA Golgi-type/AC-22* +), anti-U1RNP antibodies) suggests an increased risk of ischemia.

Blood Parameter	Value	Unit	Normal Range
Erythrocyte sedimentation rate	79	mm/h	3-23
C-reactive protein	32.1	mg/l	<5
Leukocytes	15.4	x10^9^/l	3.4 - 9.7
Neutrophils	81.7	%	44 - 72
Haptoglobin	> 2.90	g/l	0.30 - 2.20
Hemoglobin	111	g/l	138 - 175
Glucose	7.0	mmol/l	4.4 - 6.4
PV	1.15		>0.7
APTV	24.6	s	20.0 - 30.0
Creatinine	55	μmol/l	60 - 104
Glomerular filtration	110	mL/min/1.73 m^2^	>60
Cooper (S)	18.6	μmol/l	12.2 - 25.1
Cooper (dU)	0.46	μmol/dU	<1.25
Ceruloplasmin	0.32	g/l	0.20 - 0.60
Leukocyte esterase (U)	3+		Negative
Proteins (U)	negative		Negative
TSH	0.74	mIJ/l	0.35 - 4.9
T4	91	nmol/l	62.0 - 150.00
Anti-TPO	0.23	kIJ/l	≤5.61
Anti-Tg	1,3	kIJ/l	≤4.1
Antinuclear antibodies - ANA	Golgi-type (AC-22* +)	titer	<1:100
Anti-U1-RNP antibodies	154	AU/ml	Negative
Anti-MAG antibodies	negative		Negative
Antiganglioside antibodies	negative		Negative
Rheumatoid factor (RF)	< 20	IU/mL	
Anti-citrulline antibodies	< 8	U/ml	<17
Anticardiolipin antibodies-Acl-IgG	2.6	CU	<20
ANCA	negative	titer	Negative
Cholecalciferol (vit D)	108	nmol/L	>75
Cyanocobalamin (vit B12)	583	pmol/L	138 - 652
Folate acid	22.4	nmol/L	7.0 - 45.4
CYP2D6	Genotype *1/*17 (EM)		
CYP2D6	Genotype *1/*41 (IM)		

Anti-U1RNP antibodies are the hallmark of mixed connective tissue disease, but are also found in other connective tissue disorders such as systemic lupus erythematosus (SLE), idiopathic inflammatory myopathies, or primary Sjögren’s syndrome.

The fundus was normal. Chest X-ray and electrocardiogram were normal. Heart ultrasound was normal. Based on the patient’s anamnestic data and a score of 4 on the Naranjo Adverse Drug Reaction Probability (ADR) Scale, extrapyramidal syndrome was suspected as a side effect of MTX therapy. Then the immunologist switched the therapy from MTX 12.5 mg/week to sulfasalazine 500 mg/day for 12 weeks, since MTX can have prolonged effects between four and six weeks.

According to the Oromandibular Dystonia Rating Scale (OMDRS), a negligible improvement in lingual dystonia was noted 12 weeks after MTX was discontinued from therapy, but it was reintroduced due to worsening of symptoms of hidradenitis suppurativa. Then we introduced clonazepam (0.5 mg; three times daily) and baclofen (5 mg; three times daily), which led to mild improvement of lingual dystonia. Tetrabenazine therapy (12.5 mg thrice daily) further alleviated the symptoms of lingual dystonia, but the disabling lingual dyskinesias still persisted, significantly threatening the patient's quality of life and interfering with his daily activities. The lack of recovery of lingual dystonia after prolonged discontinuation of MTX, along with a clearly visible thalamic infarction, suggests that lingual dystonia could primarily be caused by thalamic infarction, and less likely a consequence of MTX neurotoxicity. Due to the lack of effect with oral therapy, in order to alleviate the symptoms of lingual dystonia, we treated a patient with the botulinum neurotoxin type A (BoNT/A) injection (20IJ) into the genioglossal muscle via submandibular and intraoral route. One month after the application of BoNT/A treatment for lingual dystonia, the patient was examined and interviewed immediately before re-injection to evaluate satisfaction and the time course of treatment effect. A comprehensive disease-specific scale, the OMDRS, was used and includes detailed assessment: (i) Examiner-rated scale (0-86 points), (ii) patient-rated questionnaire (0-84 points), and (iii) patient-rated questionnaire (Subscales of CDIP-58). OMDRS evaluates disease severity, disability, psychosocial functioning, and impact on the quality of life as well as therapeutic changes in patients with oromandibular dystonia. OMDRS were significantly reduced after administration of BoNT. Overall, BoNT treatment satisfaction was high 6 weeks later, but declined at the end of the cycle of four months of the patient's injection interval. From injection visit to a control visit 6 weeks later, investigator-rated OMDRS sum scores and sub-scores, and OMDRS scores were significantly improved compared with the first injection visit. The patient did not report adverse events. OMDRS scores indicated the re-emergence of symptoms before re-injection. Patient preferred an injection interval of < 12 weeks. Follow-up CT scan after 2 months was unchanged.

## Discussion

The patient developed lingual dystonia as an isolated symptom of chronic thalamic infarction. A potential additional cause of lingual dystonia in this patient can also be MTX therapy. DM, hypertension, smoking, alcohol, and autoimmune disease were risk factors for stroke. Although the Naranjo ADR score initially suggested a possible side effect of MTX therapy, the lack of response after withdrawal made MTX a less likely cause, and suspicion shifted to thalamic infarction as a cause of lingual dystonia. Finally, lingual dystonia should be considered if there is no other apparent cause of lingual dystonia, especially if MTX is administered orally. High-dose MTX administration, either via intravenous infusion, can cause substantial neurotoxicity [9,10]. Acute MTX-induced central neurotoxicity can present as seizure, focal neurological deficits, stroke-like episodes, posterior reversible encephalopathy syndrome, or diffuse encephalopathy, and it is dose-dependent. Only high doses of intravenous or intrathecal MTX are associated with the development of acute movement disorders, which are transient with recovery within 1-7 days from the onset of symptoms. Subacute MTX neurotoxicity typically occurs 2 to 14 days after prolonged low-dose oral or high-dose MTX. Chronic side effects such as MTX-induced leukoencephalopathy develop more slowly and ultimately result in persistent focal neurological deficits.

In general, MTX-induced extrapyramidal symptoms are extremely rare and are likely a consequence of disruption of basal ganglia connections. Mechanisms by which MTX causes leukoencephalopathy could be in its ability to dilate blood vessels with consequent cytotoxic edema and/or direct neuronal damage. A characteristic radiological features are reversible changes visible on CT as low attenuation in the white matter of both cerebral hemispheres, while MRI will typically demonstrate regions of restricted diffusion across multiple vascular territories in the centrum semiovale bilaterally, which disappear after symptom resolution [8,9]. Persistent symptoms in our patient suggest vascular occlusion rather than progressive anoxic depolarization of neurons during brain ischemia, with an increase in excitatory neurotransmitters glutamate and aspartate, that activate a number of apoptotic and necrotic pathways, resulting in neuronal dysfunction or brain death. MTX administered orally may be an additional factor that can precipitate lingual dystonia in the long term, and is less likely to trigger it directly or acutely. Despite their significance, data on clinical manifestations, prevalence, and risk factors for MTX-related neurological complications, including lingual dystonia, are scarce, and lingual dystonia has not yet been described as an isolated complication of MTX neurotoxicity.

The hypoglossal nucleus controls tongue movement and is organized with lingual protrudor motor neurons ventral and lingual retractor motor neurons dorsal. The hypoglossal motor neurons receive primarily excitatory inputs during protrusion. Although lingual dystonia is primarily due to involvement of the striatum, indirect involvement of the thalamus, cortical or subcortical pathways may lead to disinhibition that may explain the movement disorder.

An interesting hypothesis that lesions of the thalamus can cause involuntary movements was proposed by Dejerine and Roussy in 1906. Gupta and Pandey performed a literature search and reported 342 cases of thalamic poststroke movement disorders [2]. Thalamic strokes are common [2], but lingual dystonia is extremely rare following stroke [5]. The most common lesions in patients with poststroke lingual dystonia are those of the basal ganglia and subcortical white matter. There are only four cases of lingual dystonia associated with stroke of the thalamus [5]; Kim et al. reported a case of lingual dystonia following acute infarct in the right posterolateral thalamus, which occurred at the onset of symptoms and resolved four months later. However, this patient also had multiple old infarctions in the bilateral thalami and basal ganglia [6]. Pandey and Tater performed the literature search and presented 11 patients with lingual dystonia associated with structural lesions of the CNS [5]. The authors identified two more cases that link thalamic stroke and lingual dystonia; one due to left thalamic hemorrhage and one due to chronic infarcts in the left thalamus. The first patient was a 55-year-old male who had severe lingual dystonia at rest and mild on protrusion. He presented with a history of sudden-onset of slurring of speech and right-sided hemiplegia with altered behavior for 3 days. He died after 2 weeks. The second patient was a 62-year-old female who had severe tongue dystonia at rest and mild dystonia on protrusion. Her history revealed left hemiparesis and slurring of speech 2 years prior. She also had chronic ischemic lesions in the left thalamus, bilateral cerebellum, right basal ganglia, right posterior frontal and parietal areas, and left corona radiata regions [5]. In this series, the time to onset of symptoms and presentation to the hospital ranged from 4 to 24 months. Alsukhni et al. reported persistent tongue protrusion dystonia after occipitotemporal, cerebral peduncle, and bilateral thalamic infarctions [10]. The thalamus is the motor and sensory relay structure, and lesions in this location may cause abnormal involuntary movements, including delayed-onset dystonia [3-5]. It has been proposed that dystonia can be induced by disruption of the cortico-striato-pallido-thalamo-cortical loop and disinhibition of thalamocortical projections caused by blockade of GPi/STN inhibitory fibers [11,12]. According to the CT scan, as the brain MRI was not possible to perform, no ischemic lesions were described in the basal ganglia and in the subcortical white matter of the brain in our patient, in regions that are traditionally attributed to play a key role in the development of lingual dystonia. Such as a small infarction (< 15 mm) in the thalamus in our patient, indicates that even small fibers could play an important role in controlling tongue movements.

Neuroimaging studies provide more insights into structural and functional brain abnormalities underlying the complex pathophysiological mechanisms of this disorder and opening new possibilities for its objective diagnosis. Functional MRI during activity and at rest can detect the abnormalities in thalamus, basal ganglia, cerebellum, and sensorimotor cortex in various forms of isolated dystonia and point to abnormal flow of sensorimotor information within the basal ganglia-thalamo-cortical and cerebello-thalamo-cortical circuitries [13]. Functional MRI shows disintegration of neural circuits in patients with focal isolated dystonia compared to healthy subjects.

Pandey and Tater reported that overall, more left-sided than right-sided strokes were observed with poststroke lingual movement disorder. They also suggested that the poststroke lingual dystonia is responsible for the dysarthria rather than for the tongue tremor and tongue/facial weakness [5]. The left hemisphere has been reported to be dominant for tongue movements in right-handed individuals, and the majority of patients have dysarthria, and rarely tongue weakness as well [13,14]. Usually, secondary lingual dystonia develops months after the acute stroke due to neuronal reorganization.

Several pathogenic factors may explain CNS tissue damage in this patient: direct infiltration of CNS by mononuclear cells, vascular injury, and noninflammatory thickening of small vessels due to the presence of antineuronal antibodies associated with ischemia secondary to small-vessel vasculitis, revealing abnormalities in thalamus, region supplied by smaller blood vessels, where the lacunar infarcts can occur [15]. The data supplement shows risk factors for developing ischemia. Anti-Golgi antibodies (AGAs) and antibodies to U1 ribonucleoprotein (U1RNP) are important serologic biomarkers of autoimmunity and are strongly linked to various autoimmune disorders. An elevated ESR may intensity of a general atherosclerotic process and thus may be a marker for advanced atherosclerosis heralding increased risk of arterial thrombosis leading to ischemic stroke. Leukocytosis severity correlates with ischemic damage severity and outcomes. The presence of inflammatory markers raised ESR, CRP, and leukocytosis) and positive autoimmune markers (ANA) Golgi-type (AC-22* +) suggesting increased risk of ischemia. Based on studies on the vascularization identifying four major thalamic vascular territories (tuberothalamic, paramedian, inferolateral, and posterior choroidal thalamic vascular territories (PChA), we presumed that our patient had an isolated infarction in the territory supplied by the lateral posterior choroidal arteries (LPCA) originating from the distal posterior cerebral artery (PCA). Although PChA territory infarcts remain the least well-known type of thalamic infarcts, it is presumed that this territory comprises the choroid plexus of the lateral ventricle, the pulvinar, the posterior part of the dorsolateral nucleus, the lateral geniculate body, and the posterior part of the caudate nucleus, hippocampus and the mesial temporal lobe [15]. It is known that basal ganglia vascular lesions occur more frequently among patients with small-vessel cerebrovascular disease. Lingual dystonia is usually unresponsive to oral drugs but may benefit from botulinum toxin injections into the genioglossus muscle.

There are some limitations in our study. First, ischemic lesions can be missed on CT, and we may see more ischemic infarcts on MRI. Detailed evaluation of high-resolution T1-weighted and diffusion-weighted MR sequences led to the identification of microstructural abnormalities in almost all forms of isolated dystonia. Second, the ability to conclude regarding localization appears to be limited by the limited number of patients. Despite its limitations, the identification of lingual dystonia as part of a thalamic infarction in a patient receiving MTX therapy for autoimmune disease is important in determining the anatomical location of the associated ischemic lesion in the thalamus, as well as the potential inflammatory, immunological, and toxic effects of MTX therapy in terms of long-term potentiation of cerebral ischemia. This is important in identifying possible pathophysiological mechanisms of isolated lingual dystonia, which may help in identifying the therapeutic target. Future studies should be powered by advanced neuroimaging methodologies, including fMRI, which will be crucial for the objective diagnosis of lingual dystonia and for explanations of whether neural defects play a prominent role in symptom manifestation.

## Conclusions

Tongue motor function may be altered by different conditions, including vascular, inflammatory, and medication. Several pathogenic factors may cause lingual dystonia due to neuronal damage in patients on MTX therapy, due to hidradenitis suppurativa. Elevated inflammatory markers such as ERS, CRP, as well as the presence of antineuronal antibodies, are associated with ischemia. MTX can cause neuronal damage by dilating blood vessels, causing cytotoxic edema. Thalamic infarction may cause lingual dystonia by disruption of the cortico-striato-pallido-thalamo-cortical loop and disinhibition of thalamocortical projections. Temporal relationship between various conditions and lingual dystonia and the earlier proposed mechanisms, together with other cases, strongly support the hypothesis of thalamic involvement in the pathogenesis of lingual dystonia. Recognition of lingual dystonia in the setting of thalamic infarction and cerebral ischemia can be helpful in localizing lesions, identifying the etiology and pathophysiology of neurological deficits, and suggesting targets for treatment, influencing long-term outcomes. Future studies should focus on the implementation of new non-invasive methods to assess the altered functional network in lingual dystonia. Neuroimaging studies have the potential to differentiate lingual dystonia based on neuroimaging data rather than on clinical evaluation of symptoms, and to elucidate the neurophysiology, pathophysiology, and etiology of lingual dystonia. This would open up further perspectives in the development of dystonia-specific diagnostic biomarkers.
